# Safety, antitumor activity and biomarkers of sugemalimab in Chinese patients with advanced solid tumors or lymphomas: results from the first-in-human phase 1 trial

**DOI:** 10.1007/s00262-021-03102-3

**Published:** 2022-01-05

**Authors:** Jifang Gong, Junning Cao, Qingyuan Zhang, Nong Xu, Yanqiu Zhao, Baocai Xing, Zhanhui Miao, Yilong Wu, Hongming Pan, Quanli Gao, Xingya Li, Baorui Liu, Wei Li, Zhidong Pei, Hongqiang Xia, Qinzhou Qi, Hangjun Dai, Qingmei Shi, Jianxin Yang, Jin Li, Lin Shen

**Affiliations:** 1 Key Laboratory of Carcinogenesis and Translational Research (Ministry of Education/Beijing), Department of Gastrointestinal Oncology, Peking University Cancer Hospital & Institute, No. 52, Fucheng Road, Haidian District, Beijing, 100142 China; 2 Department of Medical Oncology, Fudan University Shanghai Cancer Center, Shanghai, China; 3 Department of Mammary and Lymphatic Medical Oncology, Harbin Medical University Cancer Hospital, Harbin, China; 4 Department of Medical Oncology, The First Affiliated Hospital of Zhejiang University, Hangzhou, China; 5 Respiratory Department of Internal Medicine, Affiliated Cancer Hospital of Zhengzhou University, Henan Cancer Hospital, Zhengzhou, China; 6 Department of Hepatobiliary and Pancreatic Surgery, Peking University Cancer Hospital & Institute, Beijing, China; 7 Department of Oncology, The First Affiliated Hospital of Xinxiang Medical University, Weihui, China; 8 Guangdong Lung Cancer Institute, Guangdong Provincial People’s Hospital, Guangzhou, China; 9 Department of Medical Oncology, Sir Run Run Shaw Hospital, Hangzhou, China; 10 Department of Biotherapy, Affiliated Cancer Hospital of Zhengzhou University, Henan Cancer Hospital, Zhengzhou, China; 11 Department of Oncology, The First Affiliated Hospital of Zhengzhou University, Zhengzhou, China; 12 Department of Oncology, Nanjing Drum Tower Hospital, Nanjing, China; 13 Department of Oncology, The First Bethune Hospital of Jilin University, Changchun, China; 14 Department of Oncology, Luoyang Central Hospital, Luoyang, China; 15 CStone Pharmaceuticals (Suzhou) Co., Ltd, Suzhou, China; 16 Department of Oncology, Shanghai East Hospital, Tongji University School of Medicine, No. 150, Jimo Road, Pudong New District, Shanghai, 200120 China

**Keywords:** Immunotherapy, PD-L1, Solid tumor, Sugemalimab

## Abstract

**Background:**

This first-in-human phase 1 trial is to evaluate the safety, pharmacokinetics, preliminary efficacy, and biomarkers of sugemalimab, a full-length, fully human anti-PD-L1 monoclonal antibody, in Chinese patients with advanced malignancies.

**Methods:**

Eligible patients with unresectable advanced or metastatic solid tumors or lymphomas were enrolled in phase 1a to receive sugemalimab following a modified 3 + 3 design. The primary endpoints included safety, tolerability, and the recommended Phase 2 dose (RP2D). In phase 1b, patients with 7 selected types of tumor received sugemalimab at the RP2D alone (monotherapy cohorts) or in combination with standard-of-care (SOC) chemotherapy (combination cohorts). The primary endpoint of phase 1b was investigator-assessed objective response rate (ORR).

**Results:**

As of 19 February 2020, 29 and 178 patients were treated in phase 1a and 1b, respectively. No dose-limiting toxicities were observed in phase 1a, and the RP2D of sugemalimab was determined as 1200 mg fixed dose once every 3 weeks. Sugemalimab-related adverse events (AEs) were mostly (75.9%) grade 1–2 in phase 1a. Antitumor activity was observed across dose levels with an ORR of 24.1%. In phase 1b, 15.9% and 40.4% of patients in the monotherapy and combination cohorts, respectively, reported grade 3–5 sugemalimab-related AEs. Promising efficacy was observed in all combination cohorts, with ORRs ranging from 47.6 to 75.0%. Exploratory biomarker analysis did not indicate significant differences in responses at different PD-L1 expression/tumor mutation burden levels.

**Conclusions:**

Sugemalimab was well-tolerated and showed promising antitumor activity as monotherapy or in combination with SOC chemotherapy in advanced malignancies.

This trial was registered with ClinicalTrials.gov on Oct 18, 2017, number NCT03312842.

**Supplementary Information:**

The online version contains supplementary material available at 10.1007/s00262-021-03102-3.

## Introduction

Cancer has become the leading cause of death and a major barrier to extending life expectancy worldwide in the past decade [1]. As the most populous country, China carries the most significant burden, with one-fourth of the estimated global cancer cases and deaths in 2018 occurring in the country [1, 2]. In China, cancers with poor prognosis, such as non-small cell lung cancer (NSCLC), esophageal squamous cell carcinoma (ESCC), hepatocellular carcinoma (HCC), and gastric cancer, account for more than half of all cancers diagnosed in China whereas they only comprise one-fifth of cancer incidence in developed countries [2]. Therefore, the demand for novel and effective anti-cancer therapies remains huge in China.

It has been well established that blocking the interaction between programmed death-1 (PD-1) and its ligand PD-L1 reinvigorates dysfunctional tumor-infiltrating effector T cells to overcome adaptive immune resistance and enhance antitumor activity [3–5]. Multiple immunotherapies targeting PD-1/PD-L1 have thus been investigated and demonstrated promising antitumor responses in a wide spectrum of tumors [6–16], bringing positive impacts on the treatment options and outcomes in cancer patients.

Sugemalimab is a full-length, high-affinity, fully human PD-L1 blocking, IgG4 monoclonal antibody developed using the OmniRat® transgenic rat platform. It mirrors natural IgG4 human antibody with expected pharmacokinetics (PK) profiles, which may potentially reduce the risk of immunogenicity and related toxicity in patients [17, 18]. In vitro, sugemalimab specifically binds to PD-L1, competitively blocks the binding of human PD-L1 with PD-1 and CD80, and effectively induces CD4+ T lymphocyte proliferation and enhances the production of interferon-γ and interleukin-2, suggesting the potentials in enhancing antitumor immunity [19]. In vitro studies also suggest that unlike other Fc-null PD-L1 blocking antibodies such as durvalumab, sugemalimab retains the binding to FcγR I and therefore could efficiently induce antibody-dependent cellular phagocytosis through crosslinking of PD-L1 positive tumor cells with macrophages that are prevalently present in the tumor microenvironment [19]. This mechanism may further enhance tumor antigen presentation for long-term antitumor immunity. In in vivo efficacy studies, sugemalimab significantly inhibited tumor growth in a syngeneic tumor model in which the host mice were engineered to express humanized PD-1 and implanted with MC38 tumor cells expressing human PD-L1.

This first-in-human phase 1 trial aimed to evaluate the safety and tolerability, PK, preliminary antitumor activity, and potential predictive biomarkers of sugemalimab as monotherapy and in combination with standard-of-care (SOC) chemotherapy in Chinese patients with advanced solid tumors or lymphomas.

## Methods

### Patients

Patients were recruited from 2 study centers in phase 1a and 15 study centers in phase 1b in China. Eligible patients were 18–75 years old; had histologically or cytologically confirmed unresectable, locally advanced or metastatic solid tumors or lymphomas with at least one measurable/evaluable lesion according to Response Evaluation Criteria in Solid Tumors version 1.1 (RECIST v1.1) (solid tumors) or Lugano Classification 2014 (lymphomas); progressed since previous standard anti-cancer therapy; and had an Eastern Cooperative Oncology Group performance status (ECOG PS) of 0–1. Key exclusion criteria included known primary central nervous system (CNS) tumors; prior malignancy other than those specified in phase 1b within the past 5 years; and major cardiovascular diseases. Full inclusion and exclusion criteria are listed in Supplementary materials. All patients provided written informed consent; study procedures were approved by an independent ethics committee at each study center.

### Study design

This was a multi-center, open-label, phase 1 trial, consisting of a dose-escalation phase (1a) in solid tumors or lymphomas and an expansion phase (1b) in multiple disease-specific cohorts. Following a modified 3 + 3 dose-escalation design, patients in phase 1a were treated with sugemalimab once every 3 weeks (Q3W) intravenously at doses of 3 mg/kg, 10 mg/kg, 20 mg/kg, 1200 mg fixed dose, and 40 mg/kg, for up to 24 months. Dose-limiting toxicity (DLT) was evaluated within the first 21-day treatment cycle and defined as adverse events (AEs) fulfilling a set of prespecified criteria (see Supplementary materials) that were judged by the investigator to be probably or definitely related to sugemalimab, or whose causality was undeterminable.

In phase 1b, patients were enrolled into specific disease cohorts, including cholangiocarcinoma or gallbladder carcinoma (CC/GBC) (second-line or after [≥ 2L] or patients who were unable to tolerate or refuse the standard 1L treatment), ≥ 2L HCC, ≥ 2L solid tumors with high-microsatellite instability or mismatch repair gene deficient phenotype (MSI-H/dMMR), 1L gastric adenocarcinoma or gastroesophageal junction adenocarcinoma (GAC/GEJAC), 1L ESCC, 1L non-squamous NSCLC, 1L squamous NSCLC, and 5 other disease cohorts (NK/T lymphoma, ≥ 2L ESCC, etc.) which are not included in this report due to early termination of the enrollment and/or immature results. Patients in the cohorts of CC/GBC, HCC, and MSI-H/dMMR tumors received sugemalimab monotherapy (monotherapy cohorts) while those in the cohorts of GAC/GEJAC, ESCC, non-squamous NSCLC, and squamous NSCLC received sugemalimab in combination with SOC chemotherapies (combination cohorts). In both monotherapy and combination cohorts, sugemalimab was administrated at the recommended Phase 2 dose (RP2D) determined in phase 1a. The treatment of sugemalimab continued until intolerable toxicity, disease progression, withdrawal of consent, lost to follow-up, death, or discontinuation of study. For the SOC chemotherapies, patients in the GAC/GEJAC cohort received sugemalimab with the XELOX regimen (oxaliplatin, 130 mg/m^2^, intravenously, Day 1/cycle, up to 6 cycles; capecitabine, 1000 mg/m^2^, orally, twice daily, Day 1–14/cycle, up to 6 cycles); patients in the ESCC cohort received sugemalimab with the CF regimen (cisplatin, 80 mg/m^2^, intravenously, Day 1/cycle, up to 6 cycles; 5-fluorouracil, 800 mg/m^2^/day, intravenously, Day 1–5/cycle, up to 6 cycles); patients in the non-squamous NSCLC cohort received sugemalimab with the AC regimen (pemetrexed, 500 mg/m^2^, intravenously, Day 1/cycle; carboplatin, AUC = 5, intravenously, Day 1/cycle, up to 6 cycles), with pemetrexed maintenance therapy; and patients in the squamous NSCLC cohort received sugemalimab with the PC regimen (paclitaxel, 175 mg/m^2^, intravenously, Day 1/cycle, up to 6 cycles; carboplatin, AUC = 5, intravenously, Day 1/cycle, up to 6 cycles), followed by maintenance therapy with sugemalimab.

### Endpoints

The primary endpoints of phase 1a were safety and tolerability, maximum tolerated dose (MTD), and/or RP2D. For phase 1b, the primary endpoint was the investigator-assessed objective response rate (ORR) as a single agent or in combination with chemotherapy in specific types of tumors per RECIST v1.1 (solid tumors) or Lugano Classification 2014 (lymphomas). Secondary endpoints of phase 1a included preliminary antitumor activity, PK profile, and immunogenicity. For phase 1b, the secondary endpoints included safety and tolerability, PK profile, and immunogenicity. Exploratory endpoints of both phases 1a and 1b included exploration of the pharmacodynamic profile of sugemalimab and evaluation of PD-L1 expression level as a potential predictive biomarker.

### Assessments

Safety assessments were performed throughout the study. The severity of AEs was graded according to National Cancer Institute Common Terminology Criteria for Adverse Events version 4.03. Tumor assessment for patients with solid tumors was performed by computed tomography (CT) or magnetic resonance imaging at screening within 28 days before enrollment, every 9 weeks during the 1^st^ year on study and every 12 weeks thereafter according to RECIST v1.1. Patients with lymphomas accepted a CT examination for tumor assessment at screening, and while on study every 12 weeks according to Lugano Classification 2014.

Blood samples were collected at prespecified time points to determine the concentration of sugemalimab by ELISA. Sodium heparin anticoagulated whole blood samples were collected at pre-dose of C1D1, C2D1, and C4D1 and were sent to the central lab for flow cytometry analysis of receptor occupancy (RO) by sugemalimab. A bound strategy was used to perform RO bioanalysis of peripheral CD3+ T cells (see Supplementary materials).

PD-L1 expression in tumor tissues was retrospectively assessed by immunohistochemistry in a central lab using the VENTANA PD-L1 (SP263) assay per user manual on a BenchMark autostainer. For all treatment cohorts, PD-L1 expression was scored by the percentage of membrane-stained tumor cells (TC) and immune cells (IC) at any intensity. Tumor tissue slides with less than 100 viable tumor or immune cells were considered not evaluable. PD-L1 expression in the GAC/GEJAC and ESCC cohorts was also determined by combined positive score (CPS) [20] measuring the number of PD-L1-stained tumor cells, lymphocytes, and macrophages per 100 viable tumor cells.

### Statistical analysis

The safety analysis set included patients who received at least one dose of sugemalimab. The efficacy analysis set included patients who received at least one dose of sugemalimab and had a measurable/evaluable lesion at baseline. Pharmacokinetics analysis set included patients who received at least one dose of sugemalimab and had available blood drug concentration data.

## Results

### Dose-escalation phase 1a

**Fig. 1:**
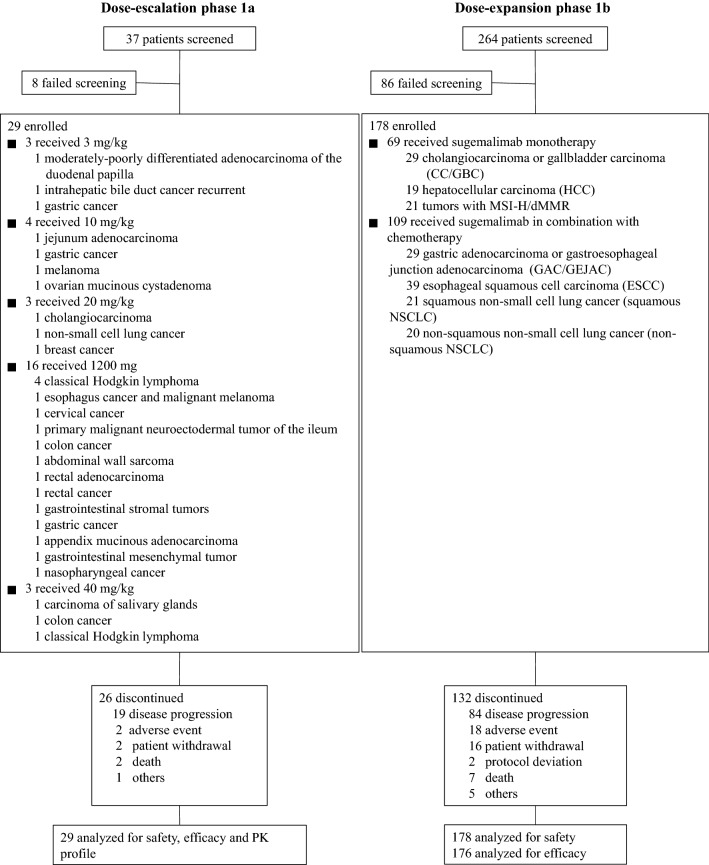
Study profile. PK, pharmacokinetics

Between 12 October 2017 and 19 February 2020, 29 patients received sugemalimab at 3 mg/kg (*n* = 3), 10 mg/kg (*n* = 4), 20 mg/kg (*n* = 3), 1200 mg fixed dose (*n* = 16), or 40 mg/kg (*n* = 3) (Fig. 1). The initial diagnosis of the patients included 22 different tumor types, with 5 patients diagnosed as classical Hodgkin’s lymphoma and the rest solid tumors. The median number of prior anti-cancer therapy regimens was 2.0 (range 0–7) (Table 1). As of the data cutoff date, 26 patients discontinued treatment, mainly due to disease progression (*n* = 19, 65.5%). Across all dosing cohorts, the median treatment duration was 126 days (range: 21–854). All patients were included in the safety and efficacy analysis set.

**Table 1:** Demographics and baseline characteristics of the enrolled patients

	Phase 1a (*N* = 29)	Phase 1b	
		Sugemalimab monotherapy (*N* = 69)	Sugemalimab in combination with chemotherapy (*N* = 109)
Age (years)	53 (23–75)	55 (25–73)	60 (23–75)
*Sex*			
Male	18 (62.1)	38 (55.1)	83 (76.1)
Female	11 (37.9)	31 (44.9)	26 (23.9)
Weight at baseline (kg)	59.2 (41.0–78.0)	58.0 (39.0–81.0)	58.4 (42.0–104.0)
*ECOG*			
0	4 (13.8)	23 (33.3)	38 (34.9)
1	25 (86.2)	45 (65.2)	71 (65.1)
Missing	0	1 (1.4)	0
*Current cancer stage*			
Stage III	0	0	1 (0.9)
Stage IIIA	0	0	2 (1.8)
Stage IIIB	0	1 (1.4)	8 (7.3)
Stage IIIC	0	0	2 (1.8)
Stage IV	26 (89.7)	58 (84.1)	83 (76.1)
Stage IVA	1 (3.4)	1 (1.4)	3 (2.8)
Stage IVB	1 (3.4)	9 (13.0)	10 (9.2)
Missing	1 (3.4)	0	0
Number of prior therapy received	2 (0–7)	1 (0–9)	0 (0–2)

Data are median (range) or n (%)

ECOG, Eastern Cooperative Oncology Group

No DLTs were reported at any dose level, and the MTD was not reached. The frequency and severity of AEs were similar among the 5 dosing cohorts. All-grade and grade ≥ 3 AEs were reported in all and 14 (48.3%) patients, respectively (Supplementary Table S1). Twenty-six (89.7%) patients experienced sugemalimab-related AEs, and the majority (75.9%) of them were grade 1–2 (Supplementary Table S2). Sugemalimab-related AEs occurred in > 20% of patients included proteinuria (*n* = 14, 48.3%), anemia (*n* = 13, 44.8%), blood bilirubin increased (*n* = 8, 27.6%), ALT increased (*n* = 7, 24.1%), AST increased (*n* = 7, 24.1%), white blood cell count decreased (*n* = 7, 24.1%), and bilirubin conjugated increased (*n* = 6, 20.7%). Grade 3 sugemalimab-related AEs were reported in 4 (13.8%) patients, and no grade 4 or 5 sugemalimab-related AEs were observed. Six (20.7%) patients experienced an SAE, including ascites, gastric hemorrhage, hepatic function abnormality, pulmonary tuberculosis, gastrointestinal neoplasm, and renal failure (*n* = 1 each), none of which was related to sugemalimab. Thirteen deaths occurred, none of which was due to AEs. Two (6.9%) patients in the 1200 mg fixed-dose group had AEs leading to sugemalimab withdrawal, which were grade 4 hepatic function abnormal and grade 3 pulmonary tuberculosis, and neither was related to sugemalimab. Seven (24.1%) patients reported immune-related AEs (irAEs) with the most common one being hypothyroidism (*n* = 4, 13.8%). No infusion-related AEs were observed.

Systemic exposure of sugemalimab was dose-proportional from 3 mg/kg to 40 mg/kg including 1200 mg fixed dose (Supplementary Table S3). Maximum serum concentration of sugemalimab (C_max_, 52.82–1278.31 μg/mL) was achieved at the end of infusion. Following a single intravenous infusion, the clearance and elimination half-life were approximately 0.141–0.250 L/day and 12.19–17.56 days, respectively. After multiple intravenous infusions, the accumulation index for C_max_ and AUC were 0.99–1.74 and 1.43–2.15, respectively. The average trough concentrations ranged from 32.7 to 418.47 μg/mL at steady state across dose levels.

Blood samples from 3 patients in the 10 mg/kg group and 4 patients in the 1200 mg group were collected for RO analysis (Supplementary Figure S1). At the time point of C2D1 pre-dose, CD3+ T cells from 7 evaluable patients showed 100% RO. Of note, compared with the 1200 mg dose group, RO from 3 patients treated with a lower dose (10 mg/kg) already showed saturation level. At the time point of C4D1 pre-dose, a consistently high level of RO (76%-100%) was shown in 3 patients.

Across all dosing cohorts, 7 patients achieved partial response (PR), resulting in an ORR of 24.1% (95% CI: 10.3, 43.5) (Supplementary Table S4), and the median DoR was 13.7 months (95% CI: 6.2, -). The median PFS was 4.8 months (95% CI: 2.2, 7.8) and the median OS was not reached (range: 2.1 to 28.1^+^ months). Notably, one patient with middle and low differentiated adenocarcinoma of ampulla initially administrated at the starting dose level of 3 mg/kg Q3W (switched to 1200 mg Q3W upon RP2D determination) experienced a durable response that lasted for almost 2 years and was still ongoing as of the cutoff date.

Based on the safety, tolerability, PK, pharmacodynamics, and preliminary antitumor efficacy data collected in phase 1a, 1200 mg fixed dose Q3W was determined as the RP2D by the safety monitoring committee.

### Dose-expansion phase 1b

Between 04 May 2018 and 19 February 2020, a total of 178 eligible patients were enrolled in phase 1b, of which 69 patients in the cohorts of CC/GBC (*n* = 29), HCC (*n* = 19) and MSI-H/dMMR tumors (*n* = 21) received sugemalimab monotherapy and 109 patients in the cohorts of GAC/GEJAC (*n* = 29), ESCC (*n* = 39), non-squamous NSCLC (*n* = 21) and squamous NSCLC (*n* = 20) received sugemalimab in combination with SOC chemotherapy (Fig. 1).

#### Monotherapy cohorts

The median age of the 69 patients was 55 years (range: 25–73) and the majority (65.2%) had an ECOG PS of 1 (Table 1). As of the cutoff date, 60 patients discontinued sugemalimab mostly due to disease progression (*n* = 44, 63.8%). The median treatment durations were 84 days (range: 21–525), 62 days (range: 21–402), and 170 days (range: 21–587) for patients in the CC/GBC, HCC, and MSI-H/dMMR tumor cohorts, respectively.

All 69 patients were included in the safety analysis. Sixty-eight (98.6%) patients experienced at least one AEs of any grade, with grade ≥ 3 AEs reported in 29 (42.0%) patients (Supplementary Table S5). Sugemalimab-related AEs were reported in 84.1% (*n* = 58) of the patients, in which 15.9% (*n* = 11) were grade 3 or 4 (Table 2). No grade 5 sugemalimab-related AEs were reported. The most common (> 20%) sugemalimab-related AEs included AST increased (23.2%), and ALT increased (21.7%) (Table 2). Five patients (7.2%) had AEs leading to withdrawal of sugemalimab. A total of 35 (50.7%) deaths occurred during the study, including 1 (1.4%) due to unknown cause (determined as an AE not related to sugemalimab by the investigator), 28 (40.6%) due to disease under study, and 6 (8.7%) due to other causes. Eighteen patients (26.1%) reported SAEs and those related to sugemalimab occurred in 7 patients (10.1%), including hepatic function abnormal (*n* = 2), anemia (*n* = 1), pancytopenia (*n* = 1), vomiting (*n* = 1), pneumonia (*n* = 1), myositis (*n* = 1), and pneumonitis (*n* = 1). Thirty-six (52.2%) patients experienced an irAE, with the most common ones being ALT increased, amylase increased, hyperthyroidism, and hypothyroidism, each in 6 patients. One infusion-related reaction (pyrexia) occurred in 1 (1.4%) patient in the CC/GBC cohort.

**Table 2:** Sugemalimab-related adverse events reported in > 10% patients and any ≥ grade 3 sugemalimab-related adverse events in dose-expansion phase 1b (*N* = 178)

Preferred term	Any grade		Grade 3–5	
	Sugemalimab monotherapy cohorts (*N* = 69)	Sugemalimab in combination with chemotherapy cohorts (*N* = 109)	Sugemalimab monotherapy cohorts (*N* = 69)	Sugemalimab in combination with chemotherapy cohorts (*N* = 109)
Number of patients with at least one sugemalimab-related AEs, n (%)	58 (84.1)	101 (92.7)	11 (15.9)	44 (40.4)
Anemia	13 (18.8)	43 (39.4)	2 (2.8)	12 (11.0)
Platelet count decreased	5 (7.2)	27 (24.8)	0	9 (8.3)
White blood cell count decreased	5 (7.2)	27 (24.8)	0	8 (7.3)
Neutrophil count decreased	4 (5.8)	24 (22.0)	0	9 (8.3)
AST increased	16 (23.2)	22 (20.2)	0	1 (0.9)
Rash	2 (2.9)	18 (16.5)	0	0
ALT increased	15 (21.7)	17 (15.6)	1 (1.4)	1 (0.9)
Blood corticotrophin increased	2 (2.9)	16 (14.7)	0	0
Amylase increased	6 (8.7)	14 (12.8)	0	4 (3.7)
Decreased appetite	1 (1.4)	14 (12.8)	0	0
Lymphocyte count decreased	0	13 (11.9)	0	2 (1.8)
Asthenia	4 (5.8)	13 (11.9)	0	2 (1.8)
Thrombocytopenia	0	10 (9.2)	0	4 (3.7)
Gamma-glutamyltransferase increased	0	10 (9.2)	0	2 (1.8)
Hypothyroidism	6 (8.7)	10 (9.2)	0	1 (0.9)
Hypertriglyceridaemia	1 (1.4)	10 (9.2)	0	1 (0.9)
Proteinuria	9 (13.0)	10 (9.2)	0	0
Fatigue	1 (1.4)	9 (8.3)	0	3 (2.8)
Hypomagnesaemia	0	8 (7.3)	0	1 (0.9)
Blood creatinine increased	1 (1.4)	8 (7.3)	0	1 (0.9)
Bilirubin conjugated increased	7 (10.1)	8 (7.3)	0	1 (0.9)
Neutropenia	1 (1.4)	7 (6.4)	0	2 (1.8)
Blood bilirubin increased	7 (10.1)	7 (6.4)	0	1 (0.9)
Hyperthyroidism	6 (8.7)	7 (6.4)	1 (1.4)	0
Hyponatraemia	2 (2.9)	6 (5.5)	2 (2.9)	3 (2.8)
Vomiting	2 (2.9)	6 (5.5)	1 (1.4)	1 (0.9)
Hepatic function abnormal	2 (2.9)	6 (5.5)	1 (1.4)	1 (0.9)
Pneumonia	1 (1.4)	6 (5.5)	1 (1.4)	0
Bone marrow failure	0	4 (3.7)	0	3 (2.8)
Blood creatine phosphokinase increased	4 (5.8)	4 (3.7)	1 (1.4)	0
Blood alkaline phosphatase increased	4 (5.8)	2 (1.8)	0	1 (0.9)
Visual impairment	0	1 (0.9)	0	1 (0.9)
Pneumonitis	1 (1.4)	1 (0.9)	1 (1.4)	1 (0.9)
Hepatitis	0	1 (0.9)	0	1 (0.9)
Febrile neutropenia	0	1 (0.9)	0	1 (0.9)
Death	0	1 (0.9)	0	1 (0.9)
Cerebral hemorrhage	0	1 (0.9)	0	1 (0.9)
Blood pressure increased	0	1 (0.9)	0	1 (0.9)
Pancytopenia	1 (1.4)	0	1 (1.4)	0
Myositis	1 (1.4)	0	1 (1.4)	0

AE, adverse event

**Fig. 2:**
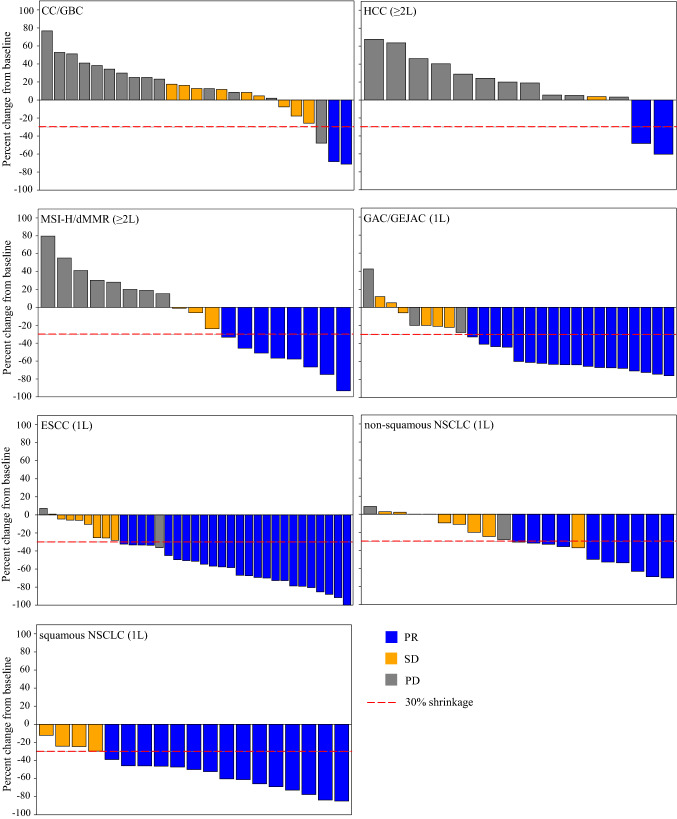
Tumor response in each disease cohort in phase 1b. Each bar represents one patient. ≥ 2L, second-line or after; 1L, first-line; CC/GBC, cholangiocarcinoma or gallbladder carcinoma; HCC, hepatocellular carcinoma; MSI-H/dMMR, solid tumors with MSI-H/dMMR phenotype; GAC/GEJAC, gastric adenocarcinoma or gastroesophageal junction adenocarcinoma; ESCC, esophageal squamous cell carcinoma; NSCLC, non-small cell lung cancer. PR, partial response; SD, stable disease; PD, progressive disease

In total, 12 of the 69 patients achieved a best overall response of PR, including 2 (1 unconfirmed) in the CC/GBC cohort, 2 (1 unconfirmed) in the HCC cohort, and 8 (2 unconfirmed) in the MSI-H/dMMR cohort, leading to ORRs of 6.9%, 10.5%, and 38.1%, respectively (Fig. 2 and Table 3). The DoRs of the 2 responders in the CC/GBC cohort were 2.8 and 8.0 months, respectively, and the responses in both patients were still ongoing as of the cutoff date, whereas the 2 responders in the HCC cohort experienced disease progression after they have responded to the treatment for 2.2 and 6.9 months, respectively. For the 8 responders in the MSI-H/dMMR cohort, the median DoR was 13.8 months (95% CI: 2.1, -). The median PFS were 2.2 months (95% CI: 2.0, 4.2), 2.1 months (95% CI: 1.4, 2.1) and 4.1 months (95% CI: 2.0, 15.8) for the CC/GBC, HCC and MSI-H/dMMR cohorts, respectively. The median OS of the CC/GBC and HCC cohorts were 11.0 months (95% CI: 6.4, 16.1) and 7.1 months (95% CI: 2.3, 18.7), respectively, while it was not reached (range: 1.3 to 19.8^+^ months) in the MSI-H/dMMR cohort.

**Table 3:** Response and survival data in each tumor type in phase 1b (*N* = 176)

	Sugemalimab monotherapy			Sugemalimab in combination with chemotherapy			
	CC/GBC (*N* = 29)	HCC (≥ 2L) (*N* = 19)	MSI-H/dMMR (≥ 2L) (*N* = 21)	GAC/GEJAC (1L) (*N* = 29)	ESCC (1L) (*N* = 37)	Non-squamous NSCLC (1L) (*N* = 21)	Squamous NSCLC (1L) (*N* = 20)
PR*, n (%)	2 (6.9)	2 (10.5)	8 (38.1)	18 (62.1)	25 (67.6)	10 (47.6)	15 (75.0)
SD, n (%)	9 (31.0)	1 (5.3)	3 (14.3)	6 (20.7)	8 (21.6)	9 (42.9)	4 (20.0)
PD, n (%)	14 (48.3)	12 (63.2)	8 (38.1)	3 (10.3)	2 (5.4)	2 (9.5)	0
NA, n (%)	4 (13.8)	4 (21.1)	2 (9.5)	2 (6.9)	2 (5.4)	0	1 (5.0)
ORR, %	6.9	10.5	38.1	62.1	67.6	47.6	75.0
DCR, %	37.9	15.8	52.4	82.8	89.2	90.5	95.0
Median DoR, months	5.4	4.5	13.8	11.3	–	8.7	6.4
(95% CI)	(2.8, 8.0)	(2.2, 6.9)	(2.1, –)	(3.9, –)	(6.2, –)	(1.8, –)	(6.2, –)
Median PFS, months	2.2	2.1	4.1	8.3	9.0	6.5	8.4
(95% CI)	(2.0, 4.2)	(1.4, 2.1)	(2.0, 15.8)	(4.8, 13.3)	(4.4, –)	(4.4, 11.7)	(8.2, –)
Median OS, months	11.0	7.1	–	17.0	–	–	–
(95% CI)	(6.4, 16.1)	(2.3, 18.7)	(14.6, –)	(12.1, –)	(9.7, –)	(10.4, –)	(13.9, –)

≥ 2L, second-line or after; 1L, first-line; CC/GBC, cholangiocarcinoma or gallbladder carcinoma; HCC, hepatocellular carcinoma; MSI-H/dMMR, solid tumors with MSI-H/dMMR phenotype; GAC/GEJAC, gastric adenocarcinoma or gastroesophageal junction adenocarcinoma; ESCC, esophageal squamous cell carcinoma; NSCLC, non-small cell lung cancer. PR, partial response; SD, stable disease; PD, progressive disease; NA, patient do not have any assessment post-baseline; ORR, objective response rate; DCR, disease control rate; DoR, duration of response; PFS, progression-free survival; OS, overall survival

^*^Response was assessed in accordance with the Response Evaluation Criteria in Solid Tumors version 1.1. Responses were unconfirmed

#### Combination cohorts

Of the 109 patients with GAC/GEJAC, ESCC, non-squamous NSCLC, or squamous NSCLC, the median age was 60 years (range: 23–75). 65.1% of them had an ECOG PS of 1 (Table 1). As of the cutoff date, 72 patients discontinued sugemalimab, with disease progression being the most frequent reason (*n* = 40, 36.7%). The median treatment durations of sugemalimab were 232 days (range: 21–523), 172 days (range: 21, 488), 315 days (range: 58–439), and 278.5 days (range: 42–509) for patients in the GAC/GEJAC, ESCC, non-squamous and squamous NSCLC cohorts, respectively.

All 109 patients experienced at least one AEs, with grade 3 or worse AEs occurring in 87 (79.8%) patients (Supplementary Table S5). Sugemalimab-related AEs were reported in 92.7% (*n* = 101) of the patients, of which 40.4% were grade ≥ 3 (Table 2). The most common (> 20%) sugemalimab-related AEs included anemia (*n* = 43, 39.4%), platelet count decreased (*n* = 27, 24.8%), white blood cell count decreased (*n* = 27, 24.8%), neutrophil count decreased (*n* = 24, 22.0%), and AST increased (*n* = 22, 20.2%) (Table 2). Thirteen (11.9%) patients experienced AEs leading to withdrawal of sugemalimab. A total of 30 (27.5%) deaths occurred during the study, including 6 (5.5%) due to AEs, 19 (17.4%) due to disease under study, and 5 (4.6%) due to other causes. Among the 6 fatal AEs, one (death) was considered related to sugemalimab only, and one (cerebral hemorrhage) was considered related to sugemalimab, pemetrexed and carboplatin; the rest were unrelated to sugemalimab. Fifty-five (50.5%) patients reported SAEs, and 24 (22.0%) patients had sugemalimab-related SAEs. The most common sugemalimab-related SAE was platelet count decreased (*n* = 6, 5.5%). A total of 64 (58.7%) patients experienced at least one irAE, the most common of which were amylase increased (*n* = 12, 11.0%) and rash (*n* = 12, 11.0%). Infusion-related reactions occurred in 7 (6.4%) patients.

Robust and durable antitumor activities were observed among the 107 patients included in the efficacy analysis set in these 4 cohorts. Two patients in the ESCC cohort, who had not reached their first post-baseline tumor assessments and were still receiving study treatment as of the cutoff date, were excluded from the efficacy analysis. In the GAC/GEJAC cohort, 18 of the 29 patients achieved PR (1 unconfirmed), resulting in an ORR of 62.1% (Fig. 2 and Table 3). The median DoR, PFS, and OS were 11.3 months (95% CI: 3.9, -), 8.3 months (95% CI: 4.8, 13.3), and 17.0 months (95% CI: 12.1, -), respectively. Among the 37 efficacy-evaluable patients in the ESCC cohort, 25 patients had PRs (5 unconfirmed). The ORR was 67.6%, with the median DoR not reached (range: 0.03^+^ to 13.3^+^ months). The median PFS was 9.0 months (95% CI: 4.4, -), and the OS ranged from 2.5 to 18.2^+^ months, with the median OS not reached. Ten of the 21 patients in the non-squamous NSCLC cohort experienced PRs (2 unconfirmed). The ORR was 47.6%. Among the responders, the median DoR was 8.7 months (95% CI:1.8, -). The median PFS and OS were 6.5 months (95% CI: 4.4, 11.7) and not reached (range: 2.7 to 16.4^+^), respectively. In the squamous NSCLC cohort, 15 of the 20 patients had PRs (1 unconfirmed), leading to an ORR of 75.0%. Among the responders, the median DoR was 6.4 months (95% CI: 6.2, -). The median PFS was 8.4 months (95% CI: 8.2, -), and the median OS was not reached (range: 1.5 to 16.7^+^ months).

### PD-L1 expression

PD-L1 expression was evaluable for 118 patients enrolled in phase 1a (1200 mg dose group only) and 1b, among which 88 (74.6%) had PD-L1 expression (TC/IC ≥ 1%). A total of 28 patients who received sugemalimab monotherapy at 1200 mg Q3W (RP2D) had TC/IC ≥ 1%, among which 8 (28.6%) patients achieved a best response of PR, while the response rate was 20.0% (*n* = 3) in the 15 patients without PD-L1 expression.

For the 26 PD-L1-evaluable GAC/GEJAC patients, the ORRs were 63.2% and 57.1% for patients with CPS ≥ 5 (*n* = 19) and CPS < 5 (*n* = 7) (Table 4), with median DoR being not reached (95% CI: 9.7, -) and 5.0 months (95% CI: 3.2, -), and median PFS being 13.3 months (95% CI: 4.4, -) and 6.3 months (95% CI: 2.0, 13.3), respectively. In the 32 PD-L1-evaluable ESCC patients, ORRs were 76.5% and 53.3% for patients with CPS ≥ 10 (*n* = 17) and CPS < 10 (*n* = 15), with median DoR being not reached (95% CI: 2.2,-) and 5.0 months (95% CI: 2.2,-), and median PFS being not reached (95% CI: 4.4,-) and 4.7 months (95% CI: 4.1, 9.0), respectively. As for the squamous and non-squamous NSCLC cohorts combined, 17 patients were evaluable for PD-L1 expression, and the ORR was 50.0% in patients with PD-L1 TC ≥ 1% and 71.4% in patients with PD-L1 TC < 1%.

**Table 4:** PD-L1 expression level and tumor responses in cohorts treated with sugemalimab in combination with chemotherapy

GAC/GEJAC (1L) (*N* = 26)*				ESCC (1L) (*N* = 32)*				Squamous and non-squamous NSCLC (1L) (*N* = 17)*	
	ORR	Median DoR, months (95% CI)	Median PFS, months (95% CI)		ORR	Median DoR, months (95% CI)	Median PFS, months (95% CI)		ORR
CPS ≥ 5	63.2% (12/19)	– (9.7, –)	13.3 (4.4, –)	CPS ≥ 10	76.5% (13/17)	– (2.2, –)	– (4.4, –)	TC ≥ 1%	50% (5/10)
CPS < 5	57.1% (4/7)	5.0 (3.2, –)	6.3 (2.0, 13.3)	CPS < 10	53.3% (8/15)	5.0 (2.2, –)	4.7 (4.1, 9.0)	TC < 1%	71% (5/7)

GAC/GEJAC, gastric adenocarcinoma or gastroesophageal junction adenocarcinoma; ESCC, esophageal squamous cell carcinoma; NSCLC, non-small cell lung cancer. 1L, first-line; CPS, combined positive score; ORR, objective response rate; DoR, duration of response; PFS, progression-free survival; TC, tumor cell

*The total number of patients were based on PD-L1 expression level-evaluable patients

## Discussion

In this first-in-human trial, we demonstrated that sugemalimab was well tolerated, without any unexpected safety issues observed, and had promising antitumor activity in Chinese patients with advanced solid tumors and lymphomas. During phase 1a dose-escalation, we demonstrated that sugemalimab can be safely administrated at doses from 3 mg/kg Q3W to 40 mg/kg Q3W. No DLTs were reported, and sugemalimab at 1200 mg intravenously Q3W was determined as the RP2D for continued evaluation in phase 1b dose-expansion. To further evaluate the safety and preliminary efficacy of sugemalimab in different treatment regimens and tumor types, phase 1b study was conducted in pre-defined tumor cohorts of CC/GBC, ≥ 2L HCC, ≥ 2L MSI-H/dMMR, etc., treated with sugemalimab as monotherapy, and in cohorts of 1L GAC/GEJAC, 1L ESCC, 1L non-squamous NSCLC, 1L squamous NSCLC, etc., treated with sugemalimab in combination with chemotherapy.

The safety profiles of sugemalimab monotherapy and combined with chemotherapy observed in this study were considered generally consistent with those reported for other anti-PD-L1/anti-PD-1 monoclonal antibody therapeutics [21]. In both phase 1a and 1b, most sugemalimab-related AEs were of grade 1 or 2 and manageable. Only 2 (6.9%) and 18 (10.1%) patients in phase 1a and 1b discontinued sugemalimab due to AEs, respectively. Two fatal AEs (death and cerebral hemorrhage) considered related to sugemalimab and/or chemotherapy by the investigators were reported in one patient each from the ESCC and non-squamous NSCLC cohorts, respectively. These two events occurred when the patients were treated outside the research centers, thus complete exclusion of their relation to sugemalimab was restricted due to the lack of detailed information.

The responses observed in phase 1a were promising and durable, with an overall ORR of 24.1% in 29 heavily pre-treated patients of 22 different types of tumors and two patients continuing to benefit from the treatment for almost 2 years. In phase 1b, the regimen of sugemalimab combined with SOC chemotherapy also showed favorable efficacy in cohorts of major cancers in the 1L setting, consistent with the mechanism that the combination of immune checkpoint inhibitors (ICIs) and chemotherapy could yield synergistic effects by regulating tumor cell/immune microenvironment interactions and improve the overall therapeutic outcome [22]. For non-squamous and squamous NSCLC, the addition of sugemalimab to carboplatin and pemetrexed or paclitaxel achieved ORRs of 47.6% and 75.0%, and PFS of 6.5 and 8.4 months, respectively, similar to those reported in phase 3 studies involving pembrolizumab plus chemotherapy [23, 24]. Clinical benefits from similar synergistic treatment effects have also been shown for GAC/GEJAC and ESCC in phase 3 trials of nivolumab/pembrolizumab plus chemotherapy [25–28]. Sugemalimab in combination with SOC chemotherapy demonstrated comparable results in these two cohorts with overall ORRs of 62.1% and 67.6%, median PFS of 8.3 months and 9.0 months, and median OS of 17.0 months and not reached, respectively. These findings support additional studies for sugemalimab plus SOC chemotherapy as a novel and effective 1L treatment option for patients with NSCLC, GAC/GEJAC and ESCC, and three double-blind, randomized phase 3 trials of sugemalimab plus SOC have thus been initiated and are currently ongoing in the mentioned three indications (NCT03789604, NCT03802591, NCT04187352). While the conclusions in this study may be approached with caution due to the limited sample size in each indication cohort, they could be further validated in these phase 3 trials.

In the exploratory biomarker analysis, the limited number of biomarker-evaluable patients in this study precludes us to draw a statistically meaningful conclusion, but we observed a trend that a higher level of PD-L1 expression may potentially lead to a higher response rate in the GAC/GEJAC cohort.

In summary, sugemalimab demonstrated a well-tolerated and manageable safety profile both as a single agent and combination therapy in Chinese patients with advanced solid tumors. The robust antitumor activity observed in the combination therapy cohorts provided solid evidence in supporting the ongoing phase 3 trials of sugemalimab plus chemotherapy as the 1L treatment for patients with advanced or metastatic NSCLC, GAC/GEJAC, and ESCC.

### Electronic supplementary material

Below is the link to the electronic supplementary material.

Supplementary file1 (PDF 386 KB)

## Data Availability

All data generated or analyzed during this study are included in this published article and its supplementary files.
