# Supplementary material for: Safety, antitumor activity and biomarkers of sugemalimab in Chinese patients with advanced solid tumors or lymphomas: results from the first-in-human phase 1 trial
Source: Cancer Immunol Immunother. 2022 Jan 5;71(8):1897–908. doi: 10.1007/s00262-021-03102-3 (PMC9293819; doi:10.1007/s00262-021-03102-3)
Supplement: Supplementary file 1 — Supplementary file1 (PDF 386 KB) [file 262_2021_3102_MOESM1_ESM.pdf]

Supplementary materials

Safety, antitumor activity and biomarkers of sugemalimab in Chinese patients with advanced solid tumors or lymphomas: results from the first-in-human phase 1 trial

CONTENTS

Supplementary Table S1. Adverse events reported in >10% patients and any ≥ grade 3 adverse events in phase 1a (*N*=29). 2

Supplementary Table S2. Sugemalimab-related adverse events reported in >10% patients and any ≥ grade 3 sugemalimab-related adverse events in phase 1a (*N*=29). 3

Supplementary Table S3. Summary statistics for serum pharmacokinetic parameters after single and multiple dosing in phase 1a (*N*=29). 4

Supplementary Table S4. Response and survival data in phase 1a in each dosing groups (*N*=29). 6

Supplementary Table S5. Adverse events reported in >10% patients and any ≥ grade 3 adverse events in phase 1b (*N*=178). 7

Supplementary Figure S1. Receptor Occupancy (RO) of sugemalimab on CD3+ T cells. 12

Supplementary methods 13

Inclusion Criteria: 13

Exclusion Criteria: 17

Definition of dose-limiting toxicities (DLT) 22

PK and PD analysis 23

Supplementary Table S1. Adverse events reported in >10% patients and any ≥ grade 3 adverse events in phase 1a (*N*=29).

Preferred Term, n (%)	Any grade	Grade 3-5
**Number of patients with at least one AEs**	29 (100)	14 (48.3)
**Anaemia**	14 (48.3)	2 (6.9)
**Proteinuria**	14 (48.3)	0
**Blood bilirubin increased**	9 (31.0)	1 (3.4)
**White blood cell count decreased**	8 (27.6)	0
**ALT increased**	7 (24.1)	0
**AST increased**	7 (24.1)	0
**Bilirubin conjugated increased**	7 (24.1)	1 (3.4)
**Decreased appetite**	6 (20.7)	0
**Cough**	5 (17.2)	0
**Nausea**	5 (17.2)	0
**Fatigue**	4 (13.8)	0
**Hypothyroidism**	4 (13.8)	0
**Blood bilirubin unconjugated increased**	3 (10.3)	0
**Blood creatine phosphokinase increased**	3 (10.3)	1 (3.4)
**Constipation**	3 (10.3)	0
**Pruritus**	3 (10.3)	0
**Rash**	3 (10.3)	0
**Upper respiratory tract infection**	3 (10.3)	0
**Vomiting**	3 (10.3)	0
**Weight decreased**	3 (10.3)	0
**Platelet count decreased**	2 (6.9)	1 (3.4)
**Bone pain**	2 (6.9)	1 (3.4)
**Neck pain**	2 (6.9)	1 (3.4)
**Hypokalaemia**	2 (6.9)	1 (3.4)
**Ascites**	1 (3.4)	1 (3.4)
**Gastric haemorrhage**	1 (3.4)	1 (3.4)
**Gastrointestinal haemorrhage**	1 (3.4)	1 (3.4)
**Gait disturbance**	1 (3.4)	1 (3.4)
**Hepatic function abnormal**	1 (3.4)	1 (3.4)
**Pulmonary tuberculosis**	1 (3.4)	1 (3.4)
**Gastrointestinal neoplasm**	1 (3.4)	1 (3.4)
**Renal failure**	1 (3.4)	1 (3.4)
**Hypertension**	1 (3.4)	1 (3.4)

AE, adverse event.

Supplementary Table S2. Sugemalimab-related adverse events reported in >10% patients and any ≥ grade 3 sugemalimab-related adverse events in phase 1a (*N*=29).

Preferred Term, n (%)	Any grade	Grade 3
**Number of patients with at least one sugemalimab-related AEs**	26 (89.7)	4 (13.8)
**Proteinuria**	14 (48.3)	0
**Anaemia**	13 (44.8)	2 (6.9)
**Blood bilirubin increased**	8 (27.6)	0
**ALT increased**	7 (24.1)	0
**AST increased**	7 (24.1)	0
**White blood cell count decreased**	7 (24.1)	0
**Bilirubin conjugated increased**	6 (20.7)	0
**Nausea**	5 (17.2)	0
**Decreased appetite**	5 (17.2)	0
**Hypothyroidism**	4 (13.8)	0
**Blood bilirubin unconjugated increased**	3 (10.3)	0
**Fatigue**	3 (10.3)	0
**Pruritus**	3 (10.3)	0
**Platelet count decreased**	1 (3.4)	1 (3.4)
**Gait disturbance**	1 (3.4)	1 (3.4)

AE, adverse event.

Supplementary Table S3. Summary statistics for serum pharmacokinetic parameters after single and multiple dosing in phase 1a (*N*=29).

Parameter (unit)	3 mg/kg (*N* = 3)		10 mg/kg (*N* = 4)		20 mg/kg (*N* = 3)		40 mg/kg (*N* = 3)		1200 mg (*N* = 16)	
	**n (n*)**	**Geometric mean (Geometric CV%)**	**n (n*)**	**Geometric mean (Geometric CV%)**	**n (n*)**	**Geometric mean (Geometric CV%)**	**n (n*)**	**Geometric mean (Geometric CV %)**	**n (n*)**	**Geometric mean (Geometric CV%)**
**Cycle 1**										
$AUC_{0\text{-}t}$ (day•µg/mL)	3 (3)	453.67 (10.95)	3 (3) [1]	2099.27 (16.31)	3 (3)	3492.67 (23.82)	3 (3)	9954.30 (12.48)	16 (16) [2]	3813.66 (22.40)
$AUC_{0\text{-}\infty}$ (day•µg/mL)	3 (3)	645.19 (19.46)	3 (3) [1]	3195.21 (5.80)	2 (2) [3]	5946.81 (16.16)	3 (3)	17869.92 (37.30)	16 (16)	6802.22 (32.98)
$AUC_{0\text{-}21d}$ (day•µg/mL)	3 (3)	453.67 (10.95)	3 (3) [1]	2099.27 (16.31)	3 (3)	3492.67 (23.82)	3 (3)	9954.30 (12.48)	15 (15)	3951.85 (17.67)
C_{max} (µg/mL)	3 (3)	52.82 (15.32)	4 (4)	257.52 (25.25)	3 (3)	349.44 (13.90)	3 (3)	1,278.31 (35.34)	16 (16)	422.59 (22.76)
T_{max} (h) [5]	3 (3)	2.05 (1.88, 7.38)	4 (4)	4.55 (1.83, 7.18)	3 (3)	2.92 (2.67, 2.97)	3 (3)	2.17 (2.10, 7.20)	16 (16)	2.06 (1.88, 3.32)
CL (L/day)	3 (3)	0.250 (31.299)	3 (3) [1]	0.200 (12.356)	2 (2) [3]	0.183 (23.120)	3 (3)	0.141 (31.558)	16 (16)	0.176 (32.978)
$t_{1/2}$ (day) [5]	3 (3)	12.19 (3.32)	3 (3) [1]	14.34 (3.59)	2 (2) [3]	15.53 (0.07)	3 (3)	16.12 (5.66)	16 (16) [2]	17.56 (6.24)
V_{ss} (L)	3 (3)	4.27 (10.54)	3 (3) [1]	3.92 (16.01)	2 (2) [3]	4.43 (33.76)	3 (3)	3.29 (26.17)	16 (16)	4.25 (24.18)
**Cycle 4**										
$AUC_{0\text{-}t}$ (day•µg/mL)	1 (1)	1017.55 (-)	1 (1) [1]	3349.00 (-)	3 (3)	5178.05 (30.86)	1 (1) [2]	10665.35 (-)	13 (13) [1, 2]	7628.74 (39.54)

Parameter (unit)	3 mg/kg (*N* = 3)		10 mg/kg (*N* = 4)		20 mg/kg (*N* = 3)		40 mg/kg (*N* = 3)		1200 mg (*N* = 16)	
	n (n*)	Geometric mean (Geometric CV%)	n (n*)	Geometric mean (Geometric CV%)	n (n*)	Geometric mean (Geometric CV%)	n (n*)	Geometric mean (Geometric CV %)	n (n*)	Geometric mean (Geometric CV%)
$AUC_{0\text{-}21d}$ (day•μg/mL)	1 (1)	1017.55 (-)	1 (1) [1]	3349.00 (-)	3 (3)	5178.05 (30.86)	0	-	11 (11) [1]	8548.90 (19.28)
$AUC_{0\text{-}tau}$ (day•μg/mL)	1 (1)	1016.78 (-)	1 (1) [1]	3348.56 (-)	3 (3)	4934.00 (39.53)	1 (1)[2]	14272.82 (-)	13 (13) [1, 2]	7897.90 (35.57)
C_{max} (μg/mL)	1 (1)	77.61 (-)	2 (2)	285.90 (31.36)	3 (3)	469.78 (14.83)	1 (1)	1,187.47 (-)	14 (14)	713.64 (25.77)
C_{min} (μg/mL)	1 (1)	32.70 (-)	2 (2)	38.06 (80.82)	3 (3)	87.56 (99.57)	1 (1)	418.47 (-)	14 (14)	211.79 (65.48)
CL_{ss} (L/day)	1 (1)	0.139 (-)	1 (1) [1]	0.233 (-)	3 (3)	0.224 (47.659)	1 (1)	0.179 (-)	13 (13) [1]	0.152 (35.574)
Accumulation index (AUC) [4]	1 (1)	2.15 (-)	1 (1)[1]	1.43 (-)	3 (3)	1.48 (11.80)	1 (1)[2]	1.58 (-)	13 (13)[1, 2]	2.00 (40.34)
Accumulation index (C_{max})	1 (1)	1.30 (-)	1 (1) [1]	0.99 (-)	3 (3)	1.34 (15.99)	1 (1)	1.03 (-)	13 (13)[1]	1.74 (43.87)

Data cutoff date: November 30, 2018

The geometric coefficient of variation % being "-" indicates that there are not enough patients to calculate the mean geometric coefficient of variation (CV%).

Abbreviations: - = not applicable; n = number of patients; n* = number of patients whose serum drug concentration is not 0, used to calculate geometric values; N = number of patients in the analysis set.

1. Only patients sampled up to at least 336 hours post dose were included. Three patients were excluded due to incomplete PK sampling;
2. One patient in 40 mg/kg treatment group,three patients in 1200 mg treatment group were sampled up to 336 hours post dose;
3. $AUC_{0\text{-}\infty}$, $t_{1/2}$, CL and V_{ss} of one patient in 20mg/kg treatment group were not included in this table.
4. Accumulation Ratio AUC = $AUC_{0\text{-}21d}$ of Cycle 4 / $AUC_{0\text{-}21d}$ of Cycle 1 if both available, otherwise, $AUC_{0\text{-}14d}$ of Cycle 4 / $AUC_{0\text{-}14d}$ of Cycle 1.
5. T_{max} was expressed as median (Min, Max); $t_{1/2}$ was expressed in arithmetic mean (standard deviation).

Supplementary Table S4. Response and survival data in phase 1a in each dosing groups (*N*=29).

	3 mg/kg (*N*=3)	10 mg/kg (*N*=4)	20 mg/kg (*N*=3)	1200 mg (*N*=16)	40 mg/kg (*N*=3)	Total (*N*=29)
**PR*, n (%)**	1 (33.3)	0	2 (66.7)	0	4 (25.0)	7 (24.1)
**SD, n (%)**	0	1 (25.0)	1 (33.3)	0	6 (37.5)	8 (27.6)
**PD, n (%)**	1 (33.3)	3 (75.0)	0	2 (66.7)	5 (31.3)	11 (37.9)
**NA, n (%)**	1 (33.3)	0	0	1 (33.3)	1 (6.3)	3 (10.3)
**ORR, % (95% CI)**	33.3	0	66.7	0	25.0	24.1 (10.3, 43.5)
**DCR, % (95% CI)**	33.3	25	100	0	62.5	51.7 (32.5, 70.6)
**Median DoR, months**	-	-	10.0	-	-	13.7
**(95% CI)**	(-, -)	(-, -)	(6.2, 13.7)	(12.0, -)	(-, -)	(6.2, -)
**Median PFS, months**	3.0	1.7	15.8	4.9	2.7	4.8
**(95% CI)**	(1.6, -)	(0.3, 3.7)	(8.3, 15.9)	(2.1, 7.8)	(2.2, 18.7)	(2.2, 7.8)
**Median OS, months**	5.6	-	-	-	18.7	-
**(95% CI)**	(3.0, -)	(2.4, -)	(13.8, -)	(5.8, -)	(7.1, -)	(9.0, -)

PR, partial response; SD, stable disease; PD, progressive disease; NA, patient do not have any assessment post-baseline; ORR, objective response rate; DCR, disease control rate; DoR, duration of response; PFS, progression-free survival; OS, overall survival.

*Responses were assessed in accordance with the Response Evaluation Criteria in Solid Tumors version 1.1.

Supplementary Table S5. Adverse events reported in >10% patients and any ≥ grade 3 adverse events in phase 1b (N=178).

Preferred Term, n (%)	Any grade		Grade 3-5	
	Sugemalimab monotherapy cohorts (N=69)	Sugemalimab in combination with chemotherapy cohorts (N=109)	Sugemalimab monotherapy cohorts (N=69)	Sugemalimab in combination with chemotherapy cohorts (N=109)
**No. of patients with at least one AEs**	68 (98.6)	109 (100)	29 (42.0)	87 (79.8)
**Anaemia**	22 (31.9)	85 (78.0)	5 (7.2)	24 (22.0)
**White blood cell count decreased**	8 (11.6)	68 (62.4)	0	17 (15.6)
**Neutrophil count decreased**	6 (8.7)	62 (56.9)	0	36 (33.0)
**Platelet count decreased**	12 (17.4)	52 (47.7)	0	15 (13.8)
**Nausea**	4 (5.8)	49 (45.0)	1 (1.4)	4 (3.7)
**Decreased appetite**	7 (10.1)	48 (44.0)	1 (1.4)	1 (0.9)
**Vomiting**	6 (8.7)	32 (29.4)	1 (1.4)	8 (7.3)
**Constipation**	5 (7.2)	31 (28.4)	0	0
**AST increased**	27 (39.1)	30 (27.5)	3 (4.3)	1 (0.9)
**Hypoalbuminaemia**	11 (15.9)	25 (22.9)	0	0
**Hyponatraemia**	6 (8.7)	25 (22.9)	2 (2.9)	8 (7.3)
**ALT increased**	24 (34.8)	24 (22.0)	3 (4.3)	1 (0.9)
**Diarrhoea**	6 (8.7)	23 (21.1)	0	0
**Hyperglycaemia**	4 (5.8)	23 (21.1)	0	2 (1.8)
**Hypertriglyceridaemia**	2 (2.9)	23 (21.1)	0	2 (1.8)
**Hypokalaemia**	5 (7.2)	23 (21.1)	2 (2.9)	7 (6.4)
**Proteinuria**	10 (14.5)	23 (21.1)	0	0

**Lymphocyte count decreased**	0	22 (20.2)	0	6 (5.5)
**Asthenia**	7 (10.1)	21 (19.3)	0	2 (1.8)
**Pyrexia**	12 (17.4)	20 (18.3)	0	0
**Gamma-glutamyltransferase increased**	7 (10.1)	19 (17.4)	3 (4.3)	2 (1.8)
**Rash**	3 (4.3)	19 (17.4)	1 (1.4)	0
**Weight decreased**	1 (1.4)	19 (17.4)	0	2 (1.8)
**Blood corticotrophin increased**	2 (2.9)	18 (16.5)	0	0
**Hypercholesterolaemia**	2 (2.9)	18 (16.5)	0	0
**Hypoaesthesia**	1 (1.4)	18 (16.5)	0	0
**Amylase increased**	7 (10.1)	16 (14.7)	0	5 (4.6)
**Thrombocytopenia**	2 (2.9)	16 (14.7)	0	5 (4.6)
**Back pain**	5 (7.2)	14 (12.8)	0	0
**Hypocalcaemia**	5 (7.2)	14 (12.8)	1 (1.4)	1 (0.9)
**Malaise**	1 (1.4)	14 (12.8)	0	0
**Cough**	9 (13.0)	13 (11.9)	0	0
**Fatigue**	2 (2.9)	13 (11.9)	1 (1.4)	3 (2.8)
**Hypochloraemia**	2 (2.9)	13 (11.9)	0	0
**Insomnia**	4 (5.8)	13 (11.9)	0	0
**Leukopenia**	1 (1.4)	13 (11.9)	0	2 (1.8)
**Productive cough**	1 (1.4)	13 (11.9)	0	0
**Pruritus**	2 (2.9)	13 (11.9)	0	0
**Blood bilirubin increased**	19 (27.5)	12 (11.0)	4 (5.8)	3 (2.8)
**Blood creatinine increased**	3 (4.3)	12 (11.0)	0	1 (0.9)
**Hyperuricaemia**	3 (4.3)	12 (11.0)	0	0
**Hypomagnesaemia**	1 (1.4)	12 (11.0)	0	1 (0.9)
**Pneumonia**	1 (1.4)	12 (11.0)	1 (1.4)	2 (1.8)
**Upper respiratory tract infection**	2 (2.9)	12 (11.0)	1 (1.4)	3 (2.8)

**Bilirubin conjugated increased**	15 (21.7)	11 (10.1)	2 (2.9)	1 (0.9)
**Dizziness**	0	11 (10.1)	0	0
**Neutropenia**	1 (1.4)	11 (10.1)	0	7 (6.4)
**Hypothyroidism**	6 (8.7)	10 (9.2)	0	1 (0.9)
**Blood alkaline phosphatase increased**	8 (11.6)	9 (8.3)	2 (2.9)	1 (0.9)
**Blood creatine phosphokinase increased**	7 (10.1)	8 (7.3)	2 (2.9)	1 (0.9)
**Hyperthyroidism**	6 (8.7)	8 (7.3)	1 (1.4)	0
**Hypertension**	4 (5.8)	8 (7.3)	1 (1.4)	2 (1.8)
**Hypophosphataemia**	2 (2.9)	8 (7.3)	0	1 (0.9)
**Weight increased**	1 (1.4)	8 (7.3)	0	3 (2.8)
**Hepatic function abnormal**	3 (4.3)	7 (6.4)	1 (1.4)	1 (0.9)
**Stomatitis**	0	7 (6.4)	0	1 (0.9)
**Abdominal pain**	7 (10.1)	6 (5.5)	2 (2.9)	0
**Haemoptysis**	1 (1.4)	6 (5.5)	0	1 (0.9)
**Abdominal distension**	7 (10.1)	5 (4.6)	1 (1.4)	0
**Hyperkalaemia**	1 (1.4)	5 (4.6)	0	2 (1.8)
**Dyspnoea**	1 (1.4)	5 (4.6)	1 (1.4)	0
**Bone marrow failure**	0	5 (4.6)	0	4 (3.7)
**Febrile neutropenia**	0	4 (3.7)	0	4 (3.7)
**Musculoskeletal pain**	0	4 (3.7)	0	1 (0.9)
**Blood pressure increased**	0	3 (2.8)	0	3 (2.8)
**Cerebral infarction**	0	2 (1.8)	0	2 (1.8)
**Blood glucose increased**	0	2 (1.8)	0	1 (0.9)
**Electrolyte imbalance**	1 (1.4)	1 (0.9)	1 (1.4)	1 (0.9)
**Death**	1 (1.4)	1 (0.9)	1 (1.4)	1 (0.9)
**Pneumonitis**	1 (1.4)	1 (0.9)	1 (1.4)	1 (0.9)
**Visual impairment**	1 (1.4)	1 (0.9)	0	1 (0.9)

**Neck pain**	1 (1.4)	1 (0.9)	0	1 (0.9)
**Blood potassium increased**	0	1 (0.9)	0	1 (0.9)
**Hypoglycaemia**	0	1 (0.9)	0	1 (0.9)
**Gastrointestinal haemorrhage**	0	1 (0.9)	0	1 (0.9)
**Impaired gastric emptying**	0	1 (0.9)	0	1 (0.9)
**Obstruction gastric**	0	1 (0.9)	0	1 (0.9)
**Oesophageal food impaction**	0	1 (0.9)	0	1 (0.9)
**Upper gastrointestinal haemorrhage**	0	1 (0.9)	0	1 (0.9)
**Abdominal infection**	0	1 (0.9)	0	1 (0.9)
**Gastroenteritis**	0	1 (0.9)	0	1 (0.9)
**Sepsis**	0	1 (0.9)	0	1 (0.9)
**Multiple organ dysfunction syndrome**	0	1 (0.9)	0	1 (0.9)
**Cerebral haemorrhage**	0	1 (0.9)	0	1 (0.9)
**Cerebrovascular disorder**	0	1 (0.9)	0	1 (0.9)
**Syncope**	0	1 (0.9)	0	1 (0.9)
**Obstructive airways disorder**	0	1 (0.9)	0	1 (0.9)
**Tracheal stenosis**	0	1 (0.9)	0	1 (0.9)
**Cholecystitis**	0	1 (0.9)	0	1 (0.9)
**Hepatitis**	0	1 (0.9)	0	1 (0.9)
**Delirium**	0	1 (0.9)	0	1 (0.9)
**Mental disorder**	0	1 (0.9)	0	1 (0.9)
**Cardiac failure**	0	1 (0.9)	0	1 (0.9)
**Haematochezia**	3 (4.3)	0	1 (1.4)	0
**Pancytopenia**	1 (1.4)	0	1 (1.4)	0
**Intestinal obstruction**	1 (1.4)	0	1 (1.4)	0
**Biliary tract infection**	1 (1.4)	0	1 (1.4)	0
**Liver abscess**	1 (1.4)	0	1 (1.4)	0

**Hepatic failure**	1 (1.4)	0	1 (1.4)	0
**Jaundice cholestatic**	1 (1.4)	0	1 (1.4)	0
**Tumour haemorrhage**	1 (1.4)	0	1 (1.4)	0
**Tumour pain**	1 (1.4)	0	1 (1.4)	0
**Anastomotic leak**	1 (1.4)	0	1 (1.4)	0
**Myositis**	1 (1.4)	0	1 (1.4)	0
**Nephrolithiasis**	1 (1.4)	0	1 (1.4)	0

AE, adverse event.

Supplementary Figure S1. Receptor Occupancy (RO) of sugemalimab on CD3+ T cells.

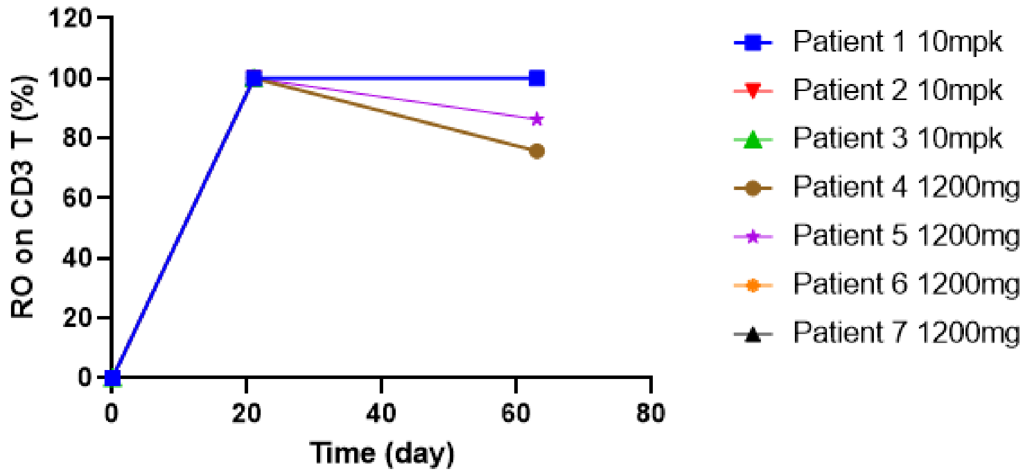

The whole blood samples from two dosing groups (10mpk and 1200mg) were collected at pre-dose (Day 1), Cycle 2 pre-dose (Day 21) and Cycle 4 pre-dose (Day 63). Molecules of Equivalent Soluble Fluorochrome data for PD-L1 RO staining and total PD-L1 staining were used to calculate the PD-L1 RO percentage for each patient in the clinical trial. At post-dose time points, RO was normalized with the baseline level at the pre-dose time point.

Supplementary methods

Inclusion Criteria:

1. Enrolled subjects must voluntarily participate in the study, fully understand, and have been informed of all aspects of the study and signed the ICF; are willing to follow and able to complete all study procedures.
2. Male or female aged between 18 and 75 (including the boundary values).
3. Enrolled subjects must have advanced or metastatic unresectable tumors that have been confirmed by histology or cytology (see the details below for the HCC enrollment criteria for HCC cohort in Phase Ib), have not received or refuse to receive standard treatment, or have failed standard treatment, or for whom standard treatment is unavailable:
   - Phase Ia: For enrolled subjects with advanced solid tumors or lymphomas, the tumor characteristics are referenced but not limited to the following types of tumors studied in Phase Ib;
   - Phase Ib: Subjects with specific types of primary tumors:

Tumor type	Inclusion Criteria
CC or GBC	1) Unresectable cholangiocarcinoma (including intra- and extra-hepatic cholangiocarcinoma, or mixed hepatocellular cholangiocarcinoma) or gallbladder cancer diagnosed by histology/cytology.<br>2) Subjects who have failed standard treatment or are unable to tolerate or refuse the standard treatment.
HCC, ≥ 2L	1) Subjects with progressive or recurrent or metastatic HCC who have previously received first-line systemic therapy for HCC and developed intolerance to treatment or progression of disease and are unsuitable for surgery or local-reginal treatment;<br>**Note: Previous systemic treatment must be chemotherapy, sorafenib or other approved first-line treatment; if a subject refuses one of these treatments, the study doctor must fully inform the subject the current treatment option, the subject must indicate refusal and the study doctor must have the written record indicating that the subject refuses to have the treatment. In addition, the subject**

	**must be fully informed by the study doctor about alternative second-line treatments such as regorafenib.**<br>2) Histologically confirmed as HCC. During the screening period of the study, subjects with imaging diagnosis only must have the diagnosis confirmed by histology prior to the initiation of the study treatment.<br>3) Child-Puge scores ≤ 7, i.e. Child-Puge A or Child-Puge B7.<br>4) Subjects with chronic HBV infection (HBV-HCC) must have HBV DNA < 500 IU/mL at screening, and HBsAg-positive patients must receive antiviral therapy according to the Guidelines for Prevention and Treatment of Chronic Hepatitis B (2015 Edition). Subjects with HCV infection (HCV-HCC), such as positive HCV RNA, must receive an approved anti-HCV standard treatment.<br>5) Albumin ≥ 2.8g/dL.
**MSI-H/dMMR, ≥ 2L**	1) Subjects with inoperable or metastatic solid tumors that fail the previous-line treatment before enrollment and do not have a satisfactory dMMR/MSI-H of alternative treatment, including but not limited to subjects with the following tumor types: colorectal cancer (Previous drugs must include fluorouracil, oxaliplatin and irinotecan), gastric cancer, pancreatic cancer, cholangiocarcinoma, endometrial cancer, etc.<br>2) Phases Ia and Ib: Subjects who have been diagnosed with MSI-H or dMMR can be screened and can receive medications when screened as eligible. Subjects are required to provide tumor and blood specimens for retrospective confirmation by a laboratory designated by the sponsor. There is no need to wait for result of retrospective confirmation for the first dose.
**GAC or GEJAC, 1L**	1) All subjects must have unresectable locally advanced or metastatic gastric or gastroesophageal junction cancer and histologically confirmed as adenocarcinoma.<br>2) Subjects must be previously untreated with systemic treatment (including HER2 inhibitors) as the primary treatment for advanced or metastatic disease.<br>3) Permitted prior treatments: Subjects with GAC or GEJAC cancer who have previously received adjuvant or neoadjuvant therapy (chemotherapy, radiotherapy or chemoradiotherapy) are allowed to be included as long as the last administration of the prior regimen occurred at least 6 months prior to the first dose of the investigational drug. If progression of disease occurs during neoadjuvant/adjuvant treatment or within 6 months after the discontinuation of the treatment, it will be considered as a first-line treatment failure.
**ESCC, 1L**	1) Histologically confirmed unresectable, recurrent or distantly metastatic locally advanced esophageal squamous cell carcinoma (excluding mixed adenosquamous carcinoma type and other pathological types).<br>2) Subjects who have received systemic treatment for locally advanced unresectable, recurrent or distantly metastatic esophageal cancer. Subjects who have received neoadjuvant/adjuvant therapy (chemotherapy, radiotherapy or chemoradiotherapy) are allowed to be included as long as the most recent treatment of the previous regimen

	occurs at least 6 months prior to the first dose of the investigational drug. If progression of disease occurs during neoadjuvant/adjuvant therapy or within 6 months of the discontinuation of the treatment, it will be considered as a first-line treatment failure.
**non-sq NSCLC, 1L**	1) Histologically or cytologically diagnosed as stage IIIB/IV non-squamous cell NSCLC (according to the cancer staging system of Union for International Cancer Control/American Joint Committee on Cancer, 7th Edition).<br>2) Subjects who are naive to systemic chemotherapy for stage IIIB/IV non-squamous cell NSCLC prior to participating in the study.<br>3) Subjects with non-metastatic disease who have received neoadjuvant and adjuvant therapy (chemotherapy, radiotherapy or chemoradiotherapy) are allowed to be included as long as the most recent treatment of the previous regimen occurs at least 6 months prior to the first dose of the investigational drug. If progression of disease occurs during neoadjuvant/adjuvant therapy or within 6 months of the discontinuation of the treatment, it will be considered as a first-line treatment failure.<br>4) About epidermal growth factor receptor (EGFR) mutations: Subjects with known EGFR mutations will be excluded (methods of assessments and results will be approved by the study site); subjects with unknown EGFR mutation status must receive prospective tests at a laboratory approved by the sponsor or study site, and subjects with an EGFR mutation will be excluded.<br>5) About ROS1, RET or ALK translocation or rearrangement: Subjects with known ROS1, RET or ALK translocation or rearrangement will be excluded (methods of assessments and results will be approved by the study site); subjects with unknown ROS1, RET or ALK translocation or rearrangement are not required to undergo the ROS1, RET or ALK test and can be screened directly.
**sq NSCLC, 1L**	1) Histologically or cytologically diagnosed as stage IIIB/IV squamous cell NSCLC (according to the cancer staging system of Union for International Cancer Control/American Joint Committee on Cancer, 7th Edition).<br>2) Subjects who are naive to systemic chemotherapy for stage IIIB/IV squamous cell NSCLC prior to participating in the study.
	3) Subjects with non-metastatic disease who have received neoadjuvant and adjuvant therapy (chemotherapy, radiotherapy or chemoradiotherapy) are allowed to be included as long as the most recent treatment of the previous regimen occurs at least 6 months prior to the first dose of the investigational drug. If progression of disease occurs during neoadjuvant/adjuvant therapy or within 6 months of the discontinuation of the treatment, it will be considered as a first-line treatment failure.

4. Subjects with ECOG performance status score of 0 or 1.

5. Subjects with at least one measurable lesion that has not been treated topically (excluding bone metastases or central nervous system [CNS] metastases as the only measurable metastatic lesions according to RECIST v1.1 for solid tumors); or subjects with at least one evaluable or measurable lesion according to the Lugano 2014 for lymphomas.

   Note: For lymphomas, subjects must have at least one measurable lesion, i.e., ≥ 1 nodal lesions (maximum diameter of > 1.5 cm), or ≥ 1 extranodal lesions (maximum diameter > 1.0 cm, e.g., liver nodular lesion); or subjects must have at least an evaluable lesion, i.e., PET/CT examinations showing that intra-lymph node or extra-nodal local uptake is higher than that of the liver and the characteristics are consistent with lymphoma manifestations.

6. Tumor tissue specimens need to be collected from the subjects for biomarker analysis. The specimens may be tumor specimens fixed with formalin and embedded in paraffin wax (10 unstained slides [applicable to subjects with confirmed MSI-H or dMMR prior to enrollment into Phase Ia and subjects in MSI-H/dMMR cohort of Phase Ib] or 7 unstained slides [applicable to subjects other than the above cohort]; fewer than the specified number of unstained slides may be submitted if approved by the medical monitor). If there are no archived tumor tissue specimens, subjects must be willing to undergo a biopsy of tumor lesion within 42 days prior to the initiation of the treatment in order to obtain the corresponding tumor specimens in quantities determined in accordance with the biopsy results.

7. Subjects who have a life expectancy of at least 3 months and safety follow-up and efficacy follow-up data can be conducted.

8. Subjects who have previously received anti-tumor therapy will only be enrolled if the toxicities from the previous treatment have returned to the baseline level (except for residual hair loss effects) or NCI CTCAE v4.03 grade ≤1.

9. Subjects with adequate organ and bone marrow functions, no serious hematopoietic dysfunction or abnormal heart, lung, liver or kidney function, or immunodeficiency under the condition that they have not received blood transfusion, Granulocyte Colony-Stimulating

Factor (G-CSF) or other medical support within14 days before receiving the investigational drug:

a) ANC ≥ 1.5×10^9/L;

b) Platelets ≥ 100×10^9/L; Phase Ib: Platelets ≥ 80×10^9/L for subjects with HCC;

c) Hemoglobin ≥ 9 g/dL;

d) Subjects with serum creatinine ≤ 1.5 × upper limit of normal (ULN) and creatinine clearance > 60 mL/min (estimated from the Cockcroft-Gault formula) can be enrolled (Note: Creatinine clearance shall be confirmed only when serum creatinine is ≥1.5 times the upper limit of the reference range);

e) Serum total bilirubin ≤ 1.5 × ULN (excluding subjects with ESCC ≥ 2L in Phase Ib; for the criteria for subjects with ESCC ≥ 2L, see the corresponding inclusion criteria for details);

f) Phase Ia: aspartate aminotransferase (AST) and alanine aminotransferase (ALT) ≤ 2.5 × ULN;

   Phase Ib (excluding subjects with ESCC ≥ 2L in Phase Ib; for the criteria for subjects with ESCC ≥ 2L, see the corresponding inclusion criteria for details): AST and ALT ≤ 2.5 × ULN; for subjects with liver cancer or liver metastases, AST and ALT ≤ 5 × ULN;

g) International normalized ratio (INR) or plasma prothrombin time (PT) ≤ 1.5 × ULN.

10. Male and female subjects of childbearing potential and their partners shall agree to use an effective method of contraception from the day of signing the ICF until 6 months after the last dose of the investigational drug.

Exclusion Criteria:

1. Subjects known to have primary CNS tumors or meningeal metastases or unstable CNS metastases (subjects with symptoms requiring hormonal therapy within 4 weeks prior to the

initiation of the study treatment, or with no imaging evidence demonstrating lesion stability for more than 4 weeks.)

2. Subjects with active autoimmune diseases or history of autoimmune diseases that may relapse (such as systemic lupus erythematosus, rheumatoid arthritis, inflammatory bowel disease, autoimmune thyroid disease, vasculitis, psoriasis, etc.) or with such risks (such as those who have undergone organ transplantation requiring immunosuppressive therapy). However, subjects with the following diseases are allowed to be enrolled for further screening: type I diabetes, hypothyroidism managed with hormone replacement therapy only, skin diseases not requiring systemic treatment (such as vitiligo, psoriasis or alopecia).
3. Subjects who are required to receive glucocorticoids (prednisone at > 10 mg/day or other similar drugs at equivalent dose) or other immunosuppressive agents within 14 days prior to the initiation of the investigational drug.

   Note: Adrenal replacement doses of ≤ 10 mg daily prednisone or equivalents are permitted in the absence of active autoimmune diseases; subjects are permitted to use topical, ocular, intra-articular, intranasal, and inhalational corticosteroids (with minimal systemic absorption); a brief course of corticosteroids for the prophylaxis (e.g., contrast dye allergy) or treatment of non-autoimmune conditions (e.g., delayed-type hypersensitivity reaction caused by contact allergen) is permitted.
4. Subjects in Phase Ib have had other malignant tumors in the past 5 years, except for radically treated skin basal cell carcinoma, breast carcinoma in situ and cervical carcinoma in situ.
5. Subjects who have received systemic anti-tumor treatments 14 days prior to the initiation of the study treatment, including chemotherapy, immunotherapy, biological therapy (tumor vaccine, cytokines, or growth factors controlling the progression of cancers).
6. Subjects who have had a major surgical procedure or radical radiotherapy within 28 days prior to receiving the study treatment, or received palliative radiotherapy within 14 days prior to receiving the study treatment, or received radioactive agents (strontium, samarium, etc.) within 56 days prior to receiving the study treatment (excluding NSCLC subjects in Phase Ib,

See the corresponding inclusion criteria for the details on the criteria for the subjects with NSCLC).

7. Subjects who have received treatment with Chinese herbal medicine or Chinese prepared medicine for anti-cancer purpose within 7 days prior to the initiation of the study treatment.
8. Subjects who have had interstitial lung disease, chemical pneumonia, allergic pneumonia, connective tissue disease pneumonia, pulmonary fibrosis, acute lung disease, etc. (except for localized interstitial pneumonia induced by radiotherapy), or uncontrolled systemic diseases, including diabetes, hypertension, etc.
9. Subjects known to have a history of human immunodeficiency virus (HIV) infection.
10. Subjects with active chronic hepatitis B or active hepatitis C (excluding HCC subjects in Phase Ib. For the criteria for the subjects with HCC, see the corresponding inclusion criteria for details). Subjects who have positive hepatitis B surface antigen (HBsAg) or hepatitis C virus (HCV) antibody during the screening period must further receive the DNA titer test (no more than 1000 copies [cps]/mL or 200 IU/mL) and HCV RNA test (exceeding the lower limit of the assay) for hepatitis B virus (HBV). Subjects can only be enrolled in the trial after they have been proven of not having active hepatitis B or hepatitis C infection requiring treatment. Subjects with the hepatitis B virus carrier and stable hepatitis B after receiving drug treatment (with a DNA titer of no more than 1000 copies [cps]/mL or 200 IU/mL) and subjects whose hepatitis C has been cured can be enrolled.
11. Subjects with active tuberculosis.
12. Subjects with any active infections requiring systemic therapy within 2 weeks prior to the initiation of the study treatment.
13. Subjects who have received organ transplantation.
14. Subjects who have received any treatment with antibodies/drugs (including anti-programmed death factor-1 [PD-1], PD-L1, etc.) that target T cell co-regulatory proteins (immune checkpoints).
15. Subjects who have developed irAE at grade ≥ 3 after receiving immunotherapy.

16. Subjects with severe allergic reactions to monoclonal antibodies (NCI CTCAE v4.03 grade greater than 3) and subjects with a history of uncontrolled allergic asthma.
17. Female subjects who are pregnant or breast-feeding; male or female subjects of childbearing potential who refuse to use an effective method of contraception; women of childbearing potential who have to undergo a pregnancy test during the screening period.
18. Subjects with a known history of alcoholism or drug abuse.
19. Subjects with major cardiovascular diseases (e.g., congestive heart failure, unstable angina pectoris, atrial fibrillation, arrhythmia, etc.): subjects who have experienced such diseases as acute myocardial infarction, unstable angina pectoris, apoplexia, or transient ischemic attack within 6 months prior to being enrolled; subjects with congestive heart failure of New York Heart Association (NYHA) Grade ≥ 2; subjects with left ventricular ejection fraction (LVEF) < 50%; and subjects with the following heart diseases:
    a) Electrocardiogram (ECG) QTc interval > 480 msec during the screening period (the QTc interval is calculated by the Fridericia formula);
    b) Right bundle branch block + left anterior half branch block or complete left bundle branch block;
    c) Subjects with congenital long QT syndrome;
    d) Subjects who have ventricular tachyarrhythmia or have a history for such a disease;
    e) Subjects with clinically significant bradycardia (< 50 beats/min);
    f) Subjects using a pacemaker;
    g) Subjects with other clinically significant heart diseases.
20. Subjects with uncontrolled pleural effusion, pericardial effusion, or ascites requiring repeated drainage within 4 weeks prior to the initiation of the study treatment (Note: Presence of a small amount of ascites that is only revealed by imaging studies is permitted).
21. Subjects with a history of mental illness.
22. Subjects who are incapacitated or have limited capacity.
23. At the discretion of the investigators, subjects with underlying conditions that may increase the risk of their receiving treatment with the investigational drug or may be confusing for the interpretation of toxic reactions and AEs.

24. In Phase Ia and Phase Ib, subjects with specific types of tumors are also required to be excluded:

Tumor type	Exclusion Criteria
**HCC, ≥ 2L**	1) Subjects with histologically proven fibrolamellar HCC, sarcomatous HCC, or HCC mixed with cholangiocarcinoma.<br>2) Subjects with a history of hepatic encephalopathy.<br>3) Viral co-infections including:<br>i. Active viral co-infections, such as HBV and HCV co-infection (detectable HBV surface antigen or HBV DNA and HCV RNA).<br>ii. Previous HBV and HCV co-infections (detectable HBV DNA, or positive HBV surface antigen with detectable HCV antibody; positive HCV RNA with resolved HBV infection: detectable HBV surface antibody, detectable HBV core antibody, undetectable HBV DNA, and undetectable HBV surface antigen).<br>4) Subjects have had esophageal or gastric variceal bleeding with in the past 6 months.
**GAC or GEJAC, 1L**	1) Subjects known to be HER2 positive. HER2 positive is defined as IHC 3+ or IHC 2+/ FISH+.<br>2) Subjects with malabsorption syndrome or unable to take medicines orally.<br>3) In addition to gastric cancer, there are clinically significant gastrointestinal abnormalities, including uncontrolled inflammatory gastrointestinal tract (e.g., Crohn's disease, ulcerative colitis, active or uncontrolled gastrointestinal bleeding, etc.).<br>4) Subjects with NCI CTCAE v4.03 Grade ≥ 2 peripheral neuropathy.<br>5) Subjects with a history of contraindications of or allergies to chemotherapy drugs (XELOX).
**ESCC, 1L**	1) Subjects with locally advanced esophageal squamous cell carcinoma undergoing radical concurrent chemoradiotherapy.<br>2) Subjects with NCI CTCAE v4.03 Grade ≥ 2 peripheral neuropathy.<br>3) Subjects with a history of contraindications of or allergies to cisplatin, carboplatin or other platinum compounds, or 5-fluorouracil.
**non-sq NSCLC, 1L**	1) Subjects with cancers histologically identified as small cell lung cancer or containing small cell components, subjects with squamous non-small cell lung cancer, and subjects with squamous cell carcinoma-based adenosquamous mixed cancer.<br>2) Subjects with EGFR-sensitive genes or with known mutations of ALK, ROS1 or RET fusion oncogenes.<br>3) Subjects with NCI CTCAE v4.03 Grade ≥ 2 peripheral neuropathy.<br>4) Subjects with a history of contraindications of or allergies to pemetrexed, carboplatin or their prophylactic use.
**sq NSCLC, 1L**	1) Subjects with cancers histologically identified as small cell lung cancer or containing small cell components, subjects with non-squamous non-small cell lung cancer, and subjects with adenosquamous mixed cancer.

	2) Subjects with NCI CTCAE v4.03 Grade ≥ 2 peripheral neuropathy.<br>3) Subjects with a history of contraindications of or allergies to taxanes or castor oil components, carboplatin or other platinum compounds.

25. In Phase Ia, the following types of subjects with cHL will also be excluded:
    a) Subjects with nodular lymphocyte-predominant Hodgkin lymphoma (NLPHL) or gray-zone lymphoma;
    b) Subjects who have received allogeneic stem cell transplantation;
    c) Subjects who have received autologous stem cell transplantation within 100 days prior to the initiation of the study treatment.

Other conditions that, in the investigators' opinion, would make subjects inappropriate to participate in this study.

Definition of dose-limiting toxicities (DLT)

All toxicities or adverse events (AEs) were graded according to the National Cancer Institute Common Terminology Criteria for Adverse Events (NCI-CTCAE) Version 4.03. Any of the following toxicities, if developed during the first cycle of the treatment and judged by the investigators to be probably or definitely related to the use of the investigational drug, or the causality is undeterminable, shall be treated as DLT:

Non-hematologic toxicities:

1. ≥ Grade 4 toxicities;
2. ≥ Grade 3 immune-related adverse events (irAEs), including immune-related pneumonia, colitis, etc.
3. Grade 3 toxicities, irrespective of the duration, except for the following: diarrhea, nausea, vomiting or abnormal electrolytes that resolved to ≤ Grade 2 within 3 days;
4. Any Grade 3 tumor flare reaction lasting for 7 days consecutively or longer (local pain, irritation or rash at known or suspected tumor foci).

Hematologic toxicities:

1. Grade 4 neutropenia lasting > 5 days;
2. Febrile neutropenia (absolute neutrophil count [ANC] < 1000/mm^3, with a single elevation of the body temperature to 38.3°C or a sustained elevation of the body temperature to 38°C for more than one hour);
3. Grade 3 neutropenia with infection;
4. Grade 3 thrombocytopenia with bleeding;
5. Grade 4 thrombocytopenia;
6. Grade 4 anemia (life-threatening).

And other toxicities of any grade that, in the judgment of the investigators and sponsor, require the subject to withdraw early from the study.

PK and PD analysis

PK parameters were presented by dose groups with geometric means, arithmetic means, coefficients of variability, general averages, and standard deviations provided, and median and range for nonparametric parameter (T_{max}). The log(C_{max})-log(Dose) and log(AUC_{tau})-log(Dose) were subjected to linear regression with the power law model and the estimated

CI of slope was used to evaluate the dose proportionality.

Molecules of Equivalent Soluble Fluorochrome (MESF) data for PD-L1 RO staining and total PD-L1 staining were used to calculate the PD-L1 RO percentage for each patient. At post-dose time points, RO was normalized with the baseline level at the pre-dose time point. Data analysis was performed using WinList software. Sodium heparin anticoagulated whole blood samples were collected at pre-dose (Day 1), Cycle 2 pre-dose (Day 21) and Cycle 4 pre-dose (Day 63). Blood samples were sent to the Central lab at ambient temperature on the collection day for FC analysis. All lab test was conducted within 48 hours after sample collection. A bound strategy was used to perform receptor occupancy (RO) bioanalysis of peripheral CD3+ T cells. In brief, a 100 µl blood sample was aliquoted into three different tubes labeled as Isotype Control tube, RO tube and Saturation tube, respectively. Blood sample in Saturation tube was firstly incubated with 250 µg/ml of CS1001 for 60 minutes at room temperature. After lysis of the red blood cells in each tube by adding 4 ml of warm whole blood lysing solution, the harvested cells were washed twice and resuspended in phosphate-buffered saline (PBS) containing 1% BSA. The samples were then incubated with Human FC Block for 10 minutes at room temperature. Two different antibody cocktails (Solution 1: IgG4 PE, CD3 PerCP, CD45 AF700, CD4 BV605; Solution 2: AHIgG4 PE, CD3 PerCP, CD45 AF700, CD4 BV605) were added into Isotype control tube (Solution 1), RO and Saturation tubes (Solution 2), respectively. Then, solution 3 (CD8 BV510) was added into all 3 tubes. All the tubes were incubated in the dark for 30

minutes at room temperature. After centrifugation, all samples were washed twice by adding 2 ml of PBS with 1% BSA. After staining, 500 µl of 1% Paraformaldehyde was added to all tubes, and stored at 2-8 ºC until acquired on BD SORP FACSCanto II.
